# Placenta Increta Complicating Persistent Cesarean Scar Ectopic Pregnancy following Failed Excision with Subsequent Preterm Cesarean Hysterectomy

**DOI:** 10.1155/2016/4071840

**Published:** 2016-06-07

**Authors:** M. F. Malik, L. R. Hoyos, J. Rodriguez-Kovacs, J. Gillum, S. C. Johnson

**Affiliations:** ^1^Department of Obstetrics & Gynecology, Detroit Medical Center, Wayne State University, Detroit, MI 48201, USA; ^2^Department of Radiology, Detroit Medical Center, Wayne State University, Detroit, MI 48201, USA

## Abstract

*Introduction.* Cesarean scar pregnancies (CSPs) are one of the rarest forms of ectopic pregnancy. Given their rarity, there is lack of consensus regarding the management and natural course of CSPs.* Case.* A 37-year-old G10 P3063 female with a history of two prior cesarean deliveries was diagnosed with her second CSP at 6 weeks and 5 days in her tenth pregnancy. The patient underwent vertical hysterotomy, excision of a gestational sac implanted in the cesarean sac, and bilateral salpingectomy via a laparotomy incision. The histopathology report confirmed immature chorionic villi. The patient returned 10 weeks later and was found to be still pregnant. Obstetric ultrasound confirmed a viable fetus of 19 weeks and 4 days of gestational age with a thin endometrium and an anteroposterior and right lateral placenta with multiple placental lakes. The patient ruptured her membranes at 31 weeks of gestation and pelvic MRI revealed an anterior placenta invading the myometrium and extending to the external serosal surface consistent with placenta increta. Following obstetric interventions, a live female infant was delivered by cesarean hysterectomy (because of placenta increta) at 32 weeks of gestation.* Conclusion.* Development of standardized guidelines for management of CSPs, as well as heightened vigilance for possible complications, is required for proper care and avoidance of potential morbidity and mortality.

## 1. Introduction

A cesarean scar pregnancy (CSP) is defined as the implantation of a fertilized ovum outside the uterus but within the fibrous tissue of a previous cesarean section scar [1]. The incidence of CSPs has been reported to occur between 1 : 1800 and 1 : 2226 of total pregnancies [1–3]. The first case of this rare form of ectopic pregnancy (EP) was described by Larsen and Solomon in 1978 [4] and was followed by overall 751 cases reported in the literature by the year 2012 [5]. The alarming increase in what was once considered a rare iatrogenic complication has been postulated to be due to two main factors: a significant rise in the number of cesarean deliveries worldwide and the increased use of transvaginal ultrasound (TVUS) for the diagnosis of early pregnancies [6]. Both elements provide not only an increase in the main risk factor for the development of a CSP, but also a means for its diagnosis.

Similar to other rare forms of EPs, there is no universal consensus among the medical community in terms of management. For this reason, it has been recommended that all cases of CSPs be reported in the literature in order to build knowledge about this rare condition with the hope of establishing treatment guidelines and recommendations [6]. Here, we report a case of a recurrent CSP managed with surgical excision that was complicated by a persistent pregnancy that survived to viability and was delivered at 32 weeks of gestation by cesarean hysterectomy because of placenta increta.

## 2. Case Presentation

A 37-year-old G10 P3063 African American female presented to the emergency department with complaints of abdominal pain and vaginal bleeding. Her obstetric history was significant for a full-term vaginal delivery followed by two full-term cesarean deliveries fifteen and five years earlier, in addition to a cesarean scar pregnancy diagnosed one year earlier. At the time of the latter, the patient underwent exploratory laparotomy, hysterotomy, and removal of the gestational sac. The uterine incision was closed with 0-vicryl in a continuous locking fashion. The pathology revealed a gestational sac with embryo which was lined by immature chorionic villi. The patient was discharged two days after surgery, had an unremarkable postoperative course, and decided to use withdrawal and condoms for contraception despite additional counseling. 

**Figure 1:**
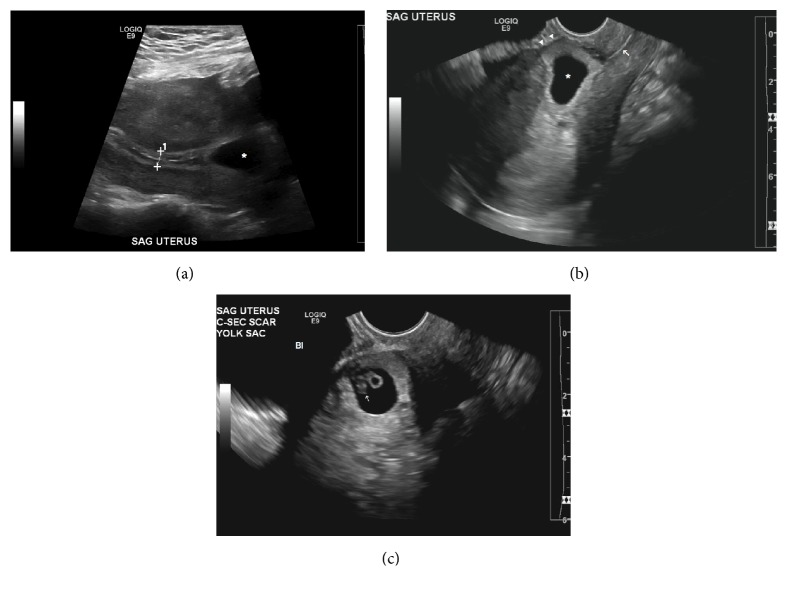
Cesarean section scar ectopic pregnancy. (a) Sagittal transabdominal image shows gestational sac (*∗*) in lower uterus inferior to trilaminar endometrium (cursors). (b) Sagittal transvaginal sonogram of gestational sac (*∗*) within the lower anterior wall and superior to the cervical canal (arrow). Note the marked thinning of the uterine wall adjacent to the sac (arrowheads). (c) Sagittal transvaginal sonogram of yolk sac and embryo (arrow) within the gestational sac (Bl: urinary bladder).

Following initial assessment in the emergency department in the present pregnancy, transvaginal ultrasound was obtained. An intrauterine pregnancy with a yolk sac but no fetal pole was found to be positioned low within the uterus. The patient was discharged at the time and instructed to continue management in the office setting. Repeat ultrasound performed one week later showed an irregular intrauterine gestational sac within the lower anterior endometrial wall within the cesarean section scar. The gestational sac has a mean internal diameter of 21 mm, corresponding to 7-week size. A yolk sac and an embryo are within the gestational sac. The embryo has a crown-rump length of 7 mm, corresponding to 6.5 weeks of gestational age. Exuberant vascularity surrounds the gestational sac wall on color Doppler. The myometrial wall anterior to the gestational sac was thinned to 1 mm (Figure 1).

The patient underwent a repeat exploratory laparotomy via a vertical hysterotomy incision made above the lower transverse uterine scar due to abundant adhesions found at this level. The products of conception were identified and excised with the remainder of the uterine cavity being suctioned. The hysterotomy was closed using 2 layers of 0-vicryl. Bilateral salpingectomy was also performed as the patient desired permanent sterilization. The pathology of the submitted products of conception reported immature chorionic villi and implantation site tissue. The patient had an uneventful postoperative course and was discharged four days after surgery. There was no *β*-hCG value or ultrasound performed in the postoperative period at this point.

**Figure 2:**
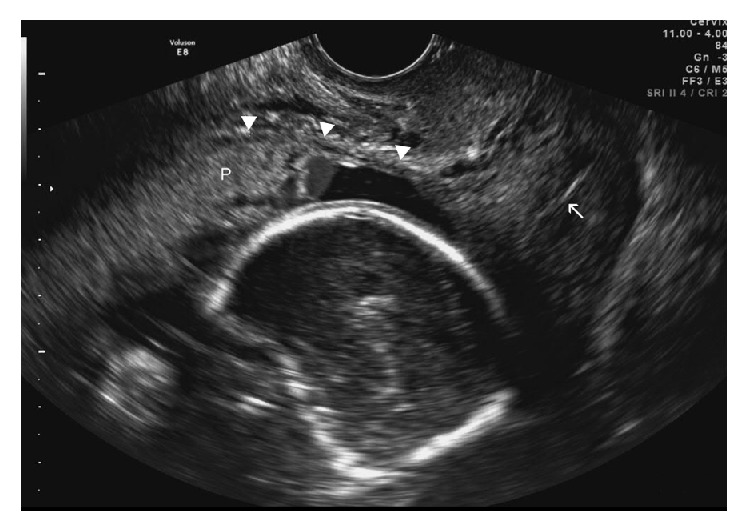
Sagittal transvaginal sonogram performed 3 months later shows intrauterine pregnancy with 19-week sized fetus. Thinned lower anterior uterine wall (arrowheads) and contiguous placenta (P) are suspicious for placenta accreta (arrow: cervical canal).

The patient had a follow-up appointment in the office one week following the surgery which was uneventful. Ten weeks later, the patient returned to the office with complaints of abdominal pain. Physical examination revealed a suprapubic mass compatible with an 18-week pregnancy with positive fetal heart tones. Formal obstetric ultrasound revealed a viable fetus at 19 weeks and 4 days of gestation with no anatomical defects (Figure 2). The lower uterine segment myometrium was very thin and the placenta was found to be anteroposterior and right lateral, with multiple placental lakes in the fundal and lower uterine segment area. There was increased vascularity in the lower segment and laterally, a finding suspicious for placenta accreta. The placental findings were confirmed on subsequent ultrasound performed eight weeks later.

**Figure 3:**
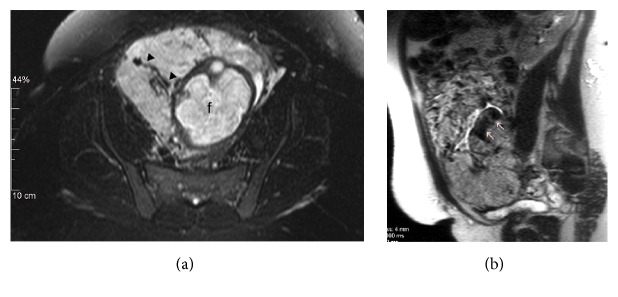
MRI of the pelvis without contrast performed 11 weeks later. (a) Transverse T2 fat suppressed image demonstrates enlarged intraplacental vessels (arrowheads) (f: fetal brain). (b) Sagittal T2 HASTE image shows thin uterine wall with dark intraplacental bands (arrows).

At 31 weeks of gestation, the patient presented with preterm premature rupture of membranes. In addition to the typical obstetric interventions involving steroids, magnesium sulfate, and latency antibiotics, pelvic MRI was obtained, which revealed an anteriorly located placenta invading the myometrium and extending to the external serosal surface. There appeared to be focal interrupted uterine serosa with tissue bulging out focally and extending to the proximal inferior vena cava with no invasion of the urinary bladder (Figure 3). With a diagnosis of placenta increta, a team of physicians and surgeons was assembled and the patient underwent an uneventful cesarean hysterectomy at 32 weeks and 5 days of gestational age. The patient delivered a viable male infant weighing 1585 grams with Apgar scores of 8 and 9 at one and five minutes, respectively. The baby remained in the NICU for 28 days due to prematurity. The patient had an uneventful postoperative course and was discharged 6 days after her surgery. In addition to a third-trimester placenta with no acute chorioamnionitis, the pathology report described a uterus with placenta increta and placental tissue extending close to the uterine serosal surface.

## 3. Discussion

We report a unique case of recurrent CSP managed with surgical excision. The case was complicated by persistence of a likely coexisting twin or heterotopic pregnancy to the point of viability and placenta increta, resulting in the delivery of a live neonate at 32 weeks of gestation via cesarean hysterectomy.

The first issue that we would like to highlight is the “persistence” of pregnancy in this patient's second presentation of CSP, despite surgical excision. Several possibilities arise as to how this could happen, considering that the pathology report identified chorionic villi and implantation site tissue. The first is that the patient could have presented with a dichorionic diamniotic twin pregnancy, in which one pregnancy was removed. The other possibility is that of a heterotopic pregnancy, in which an intrauterine pregnancy was removed surgically while a second CSP persisted. Both of these theories arise from the assumption that a second pregnancy was missed on the early six-week ultrasound report. We suspect that the pregnancy extracted was most likely an intrauterine pregnancy and the persistent pregnancy was implanted in the cesarean scar, as the placenta was found to be adherent to the anterior wall of the uterus. Doubilet and Benson's study showed that early ultrasounds obtained at 5.0–5.9 weeks frequently (14%) undercount multiple gestation instances [7]. A less likely yet possible differential for the persistent pregnancy is that the CSP that was diagnosed may have been incompletely excised. The initial first-trimester ultrasound reported the CSP and an empty endometrial cavity with a trilaminar pattern, indicating that there was no other intrauterine pregnancy seen. In addition, the pathology report did not visualize a gestational sac as reported in the patient's first CSP.

The exact pathophysiology of CSP remains unknown. However, it has been theorized that a microscopic dehiscent tract created as a result of trauma during a previous uterine surgery in addition to altered biochemical processes and low oxygen tension at the scar site may be involved [2, 8–10]. This abnormal tract may be used by the blastocyst to enter the myometrial wall [2, 9] and the low oxygen tension demonstrated during in vitro studies may allow cytotrophoblastic invasion and placental growth within the scarred area [10].

While CSPs were originally described as distinctly different from intrauterine pregnancies with placenta accreta [1], recent data suggests that they may be on a continuum of morbidly adhering placentas (MAPs) [11]. The latter occurs when there is abnormal invasion of the placenta to the myometrium with the gestational sac growing towards the uterine lumen, while a CSP occurs as an ectopic pregnancy that is implanted entirely within the cesarean scar and is surrounded by myometrium and fibrotic tissue [12]. Both entities are similar with just the site and level of invasion being different [5, 13]. However, it has been postulated that expectantly managed CSPs or those missed on diagnosis during the first trimester may have evolved into pregnancies with MAPs [11, 13]. In a recent case series of ten cases of CSP in which the patients decided to continue with the pregnancy, nine out of ten patients were able to deliver live neonates between 32 and 37 weeks of gestation [12]. All patients underwent cesarean hysterectomy at the time of delivery and all ten cases had a histopathologic diagnosis of placenta percreta, thereby validating the hypothesis that CSPs may be precursors to cases of MAPs [12]. In our case, the final pathology showed evidence of placenta increta, where villi penetrated into the depths of the myometrium although an influence of the attempted excision of the pregnancy on this abnormal invasion cannot be completely ruled out.

It may seem that not all types of CSPs are the same, and while some of them may be precursors of MAPs the others may be associated with an increased risk of rupture with its obvious dreadful clinical consequences. Vial et al. [14] categorized two types of CSPs based on their implantational characteristics. In the first type, like our case and where an argument for a precursor to MAPs can be made, there is implantation of the amniotic sac on the scar with progression of the pregnancy into the cervicoisthmic space and intrauterine cavity. The aforementioned allows for expectant management and if desired may allow for a viable birth. The second type involves deep implantation in the cesarean scar defect with progression towards rupture and bleeding. In this particular case, there is a risk of uterine rupture with life-threatening hemorrhage and the patient should be made aware and counseled on pregnancy termination [14].

In terms of diagnosis, it appears that transvaginal ultrasound remains the gold standard in early gestation as in the sagittal orientation it may be used to clearly identify a uterine cavity with an empty cervical canal, thus differentiating cesarean scar from a cervical ectopic pregnancy [1, 15]. Often the anterior uterine wall appears very thin and the pregnancy is seen bulging through and reaching close to the bladder [3]. Additionally, three-dimensional Doppler imaging has been found to be a useful tool which may identify the neovascularization characteristics associated with this particular type of ectopic pregnancy [16]. Early diagnosis provides advantages including the potential of allowing early interventions with prevention of complications such as uterine rupture, hemorrhage, need for hysterectomy, and maternal morbidity [17], which could help preserve the patient's fertility [14].

Since there is no universal standard for treatment of CSPs, a wide array of treatment options have been reported in the literature including both medical and surgical alternatives [15]. In either case, the goal remains to be the removal of the gestational sac while maintaining the patient's fertility. Medical management options have included the use of local and/or systemic methotrexate, uterine artery embolization (UAE), or the combination of UAE with local methotrexate while following the trend of *β*-hCG levels to assess for treatment resolution [8, 15]. Surgical options have included laparoscopy, dilatation and curettage, which was associated with severe maternal morbidity, laparotomy with wide local excision, and hysterectomy in cases with complications [6, 15]. It has been postulated that medical management is preferred over surgical management, with the use of transvaginal local methotrexate being the optimal method of treatment [2]. Less commonly, patients may choose to be managed expectantly which has been associated with diverse outcomes in the literature. In one study, out of six patients that chose expectant management, three resulted in uterine rupture and the need for hysterectomy [6], while, in another, two out of four cases managed expectantly ended in an emergent hysterectomy [3]. A larger case series of expectant management of seven CSPs noted eight live neonates and one fetal demise [18]. Out of these cases, one patient had uterine rupture at 38 weeks of gestation, two patients developed placenta accreta, and four pregnancies were uneventful with early cesarean sections at 36 weeks. The results of these case series were more promising and recommendations were made for an early cesarean section with a possible cesarean hysterectomy in the presence of complications such as placenta accreta, uterine rupture, and/or hemorrhage [18]. Regardless, it is recommended that the patient be presented with all the information available regarding her particular case and be given the opportunity to decide on the management of her pregnancy [3, 19].

In terms of future fertility potential and risk of recurrence, a few recent follow-up studies have been performed. In one recent review from Paris, twenty-nine women with CSP were successfully treated. Out of the twenty-four women attempting to become pregnant, twenty women conceived with intrauterine pregnancies and one developed a recurrent cesarean scar pregnancy. Thirteen out of the twenty intrauterine pregnancies appeared normal, nine were delivered by cesarean section, and the other 7 ended up in spontaneous abortions [20]. Another study from Chengdu, China, in which 189 women treated for CSP were followed up, a total of 32 women were reported to conceive again out of whom five experienced a recurrent CSP, thus giving a recurrence rate of 15.6% [21].

While there is no known prevention for CSP, preventative measures may include decreasing the rate of cesarean sections and increasing the rate of vaginal births after cesarean deliveries [13]. Suggestions have also been made for increased research into actual surgical techniques for cesarean sections such as single- versus double-layered uterine closure in order to help prevent future CSPs [13, 15, 19]. In addition, repair of the scar after the removal of a CSP also seems to be key for the prevention of another CSP [15]. It has also been suggested that all women with a cesarean scar have transvaginal ultrasounds done in the nonpregnant state in order to assess the uterine wall integrity and help in patient counseling regarding the possibility of a CSP in the future [22].

The case presented here raises several issues relevant to CSPs including their relationship to the spectrum of disease involving MAPs, their surgical management upon presentation, risk of recurrence, and role for expectant management. We recommend that if an embryo is fully visualized on ultrasound prior to excision and is not reported on the pathology report, then follow-up serial ultrasounds with trending *β*-hCG levels need to be performed and patient follow-up should be strongly encouraged in order to assess the successfulness of the pregnancy excision. In addition, heightened vigilance for rupture and the presence of placenta accreta should be present during the pregnancy, in case the patient prefers expectant management. Lastly, in the case of surgical management, the uterine scar itself should be adequately explored for assessing the underlying pregnancy.
